# TET1 exerts its anti-tumor functions via demethylating DACT2 and SFRP2 to antagonize Wnt/β-catenin signaling pathway in nasopharyngeal carcinoma cells

**DOI:** 10.1186/s13148-018-0535-7

**Published:** 2018-08-03

**Authors:** Jiangxia Fan, Yan Zhang, Junhao Mu, Xiaoqian He, Bianfei Shao, Dishu Zhou, Weiyan Peng, Jun Tang, Yu Jiang, Guosheng Ren, Tingxiu Xiang

**Affiliations:** Chongqing Key Laboratory of Molecular Oncology and Epigenetics, the First Affiliated Hospital of Chongqing Medical University, Chongqing, China

**Keywords:** *TET1*, Tumor suppressor, Nasopharyngeal carcinoma, Wnt pathway, Demethylation

## Abstract

**Background:**

TET1 is a tumor suppressor gene (TSG) that codes for ten-eleven translocation methyl cytosine dioxygenase1 (TET1) catalyzing the conversion of 5-methylcytosine to 5-hydroxy methyl cytosine as a first step of TSG demethylation. Its hypermethylation has been associated with cancer pathogenesis. However, whether TET1 plays any role in nasopharyngeal carcinoma (NPC) remains unclear. This study investigated the expression and methylation of TET1 in NPC and confirmed its role and mechanism as a TSG.

**Results:**

TET1 expression was downregulated in NPC tissues compared with nasal septum deviation tissues. Demethylation of TET1 in HONE1 and HNE1 cells restored its expression with downregulated methylation, implying that TET1 was silenced by promoter hypermethylation. Ectopic expression of TET1 suppressed the growth of NPC cells, induced apoptosis, arrested cell division in G0/G1 phase, and inhibited cell migration and invasion, confirming TET1 TSG activity. TET1 decreased the expression of nuclear β-catenin and downstream target genes. Furthermore, TET1 could cause Wnt antagonists (DACT2, SFRP2) promoter demethylation and restore its expression in NPC cells.

**Conclusions:**

Collectively, we conclude that TET1 exerts its anti-tumor functions in NPC cells by suppressing Wnt/β-catenin signaling via demethylation of Wnt antagonists (DACT2 and SFRP2).

**Electronic supplementary material:**

The online version of this article (10.1186/s13148-018-0535-7) contains supplementary material, which is available to authorized users.

## Background

The worldwide incidence and mortality of nasopharyngeal carcinoma (NPC) is very low, but it is high in southern China [1, 2]. Radiation therapy is currently the primary treatment in the earlier stage, and combined with chemoradiotherapy in the late stage, but distant metastasis and recurrence are frequent [3, 4]. As NPC is regulated by genetic and epigenetic factors [5, 6], biomarkers would help to improve treatment and outcomes. In NPC, many tumor suppressor genes (TSGs), such as *PCDH20* [7], *WIF1* [7, 8], *RASSF1* [9], *ADAMTS18* [10], *PTPRG* [11], *CDH4* [12], *CDH11* [13], *SOX11* [14], and *DACT2* [15], are silenced by hyper-methylation. Some are associated with Wnt/β-catenin pathway activation [7, 8, 13, 15, 16].

The ten-eleven translocation (TET) proteins, TET1, TET2, and TET3 are highly active DNA cytosine oxygenases that maintain TSGs in an unmethylated state by conversion of 5-methyl cytosine (5mC) to 5-hydroxymethyl cytosine (5hmC) or by competition with DNA methyltransferases resulting in passive demethylation [17, 18]. Its C-terminal region is the catalytic domain, and the N-terminal region has a conserved CXXC domain [19], which identifies cytosine. TET1 contains three nuclear localization signals, indicating potential activity in the nucleus [20]. The *TET1* gene is located at chromosome 10q21.3, and it was first described in a patient with acute myeloid leukemia associated with a chromosome translocation [21, 22]. *TET1* is active as a TSG in breast [23], colon [24], gastric [25], prostate [26], hepatocellular [27], and renal carcinoma [28]. Its hyper-methylation has been associated with cancer pathogenesis. Li et al. showed that TET1, TET2, and TET3 are highly expressed in normal tissues, but only TET1 is downregulated in nasopharyngeal carcinoma cells [29]. Therefore, this study investigated the expression and methylation of TET1 in NPC and confirmed its role as a TSG. TET1 catalyzed several TSG demethylations to renew their expression, and suppressed Wnt/β-catenin pathway. Thus, *TET1* and its candidate target genes all are potential NPC biomarkers.

## Methods

### Tumor cell lines and tumor samples

The HNE1 and HONE1 nasopharyngeal carcinoma cell lines were obtained from Prof. Qian Tao, the Chinese University of Hong Kong, Hong Kong, China. The cells were maintained in RPMI 1640 (Gibco BRL, MD, USA) supplemented with 10% fetal bovine serum (FBS; PAA Laboratories, Linz, Austria), 100 U/ml penicillin (Gibco-BRL), and 100 μg/ml streptomycin (Gibco-BRL) at 37 °C in humidified air with 5% CO_2_. Normal nasal tissues were obtained from the patients of nasal septum deviation (NSD); surgical margin tissues and nasopharyngeal carcinoma tissues were obtained from surgical patients treated at the Otolaryngology Surgery Department of the First Affiliated Hospital of Chongqing Medical University.

### DNA and RNA extraction

Genomic DNA was extracted from cell lines and NPC tissues using a QIA amp DNA Mini Kit following the manufacturer’s instructions (Qiagen, Hilden, Germany). Total RNA was extracted from cell lines and NPC tissues using TRIzol reagent (Invitrogen, Carlsbad, CA, USA). Total DNA and RNA were quantified by gel electrophoresis. Samples were stored at − 80 °C until used.

### 5-aza-2′-deoxycytidine (*Aza*) and (trichostatin A) *TSA* treatments

Aza and TSA treatments were performed as described previously [30, 31]. HNE1 and HONE1 cells were treated with final concentration 10 μmol/l Aza (Sigma-Aldrich, Steinheim, Germany) for 3 days with or without 100 nmol/l TSA (Sigma-Aldrich) for another 24 h.

### Semi-quantitative RT-PCR and quantitative real-time PCR (qRT-PCR)

Semi-quantitative RT-PCR was performed with a 10 μl reaction mixture containing 2 μl cDNA using Go-taq (Promega, Madison, WI, USA). β-actin was amplified as the control and 32 cycles for TET1 and target genes. The primer sequences are listed in Table 1. qPCR of TET1 in NPC tissues and cell lines were normalized against β-actin. qRT-PCR was using SYBR® Green PCR Master Mix (Thermo Fisher Scientific, Hong Kong, China) in the HT7500 system (Applied Biosystems).

**Table 1:** List of primers used in this study

PCR	Primer	Sequence (5′-3′)	Product size (bp)	PCR Cycles	Annealing temperature (°C)
RT-PCR	*TET1F*	AGGACCAAGTGTTGCTGCTGT	219	32	55
	*TET1R*	ATCACAGCAGTTGGACAGTGG			
	*β-actinF*	TCCTGTGGCATCCACGAAACT	315	23	55
	*β-actinR*	GAAGCATTTGCGGTGGACGAT			
qRT-PCR	*DKK1F*	CTGCATGCGTCACGCTATGT	161		60
	*DKK1R*	AGGTGGTTCTTCTGGAATAC			
	*DKK2F*	ACCCGCTGCAATAATGGAATC	99		60
	*DKK2R*	ATGGTTGCGATCTCTATGCCG			
	*Snail1F*	GAGGCGGTGGCAGACTAG	159		60
	*Snail1R*	GACACATCGGTCAGACCAG			
	*DKK3F*	CACCCTCAATGAGATGTTCC	161		60
	*DKK3R*	TGGTCTCATTGTGATAGCTG			
	*DACT1F*	CTGGAGGAGAAGTTCTTGGA	161		60
	*DACT1R*	TCCAGGTGCTCTTCAGATGT			
	*DACT2F*	AGCCGTGGGGCACATTCTG	173		60
	*DACT2R*	CCAGGTCCTGCCGATACTTG			
	*SFRP1F*	ACGAGTTGAAATCTGAGGCCATC	197		60
	*SFRP1R*	ACAGTCAGCCCCATTCTTCAG			
	*SFRP2F*	ATCCTGGAGACCAAGAGCAAGAC	142		60
	*SFRP2R*	TGACCAGATAGGGCGCGTTGATG			
	*WNT5BF*	AAATGCCACGGCGTCTCG	163		60
	*WNT5BR*	GGGTGAAGCGGCTGTTGA			
	*WNT3F*	ACGAGAACTCCCCCAACTTT	170		60
	*WNT3R*	GATGCAGTGGCATTTTTCCT			
	*WNT5AF1*	CGGTGTACAACCTGGCTGATG	101		60
	*WNT5AR1*	CACCTTGCGGAAGTCTGCC			
	*WNT7AF*	CTGGAACTGCTCTGCACTGGGA	129		60
	*WNT7AR*	GTACAGGCAGCTGTGATGGCGT			
	*WNT7BF*	TTTGGCGTCCTCTACGTGAAG	145		60
	*WNT7BR*	CCCCGATCACAATGATGGCA			
	*EcadF*	TACACTGCCCAGGAGCCAGA	103		60
	*EcadR*	TGGCACCAGTGTCCGGATTA			
	*NcadF*	CGAATGGATGAAAGACCCATCC	174		60
	*NcadR*	GGAGCCACTGCCTTCATAGTCAA			
	*β-actinF1*	GTCTTCCCCTCCATCGTG	113		60
	*β-actinR1*	AGGGTGAGGATGCCTCTCTT			
MSP	*TET1m4*	GTCGGTAGGCGTTTTTCGC	173	40	60
	*TET1m8*	CCCAACTCACCGCTAACCG			
	*TET1u4*	GAGTTGGTAGGTGTTTTTTGT	175	40	58
	*TET1u8*	CCCAACTCACCACTAACCA			
	*DKK1m1*	ATTTTGTAGTCGAATCGGTAC	127	40	60
	*DKK1m2*	CCGAATAACTCCCGCTACG			
	*DKK1u1*	TGATTTTGTAGTTGAATTGGTAT	131	40	58
	*DKK1u2*	ACCCAAATAACTCCCACTACA			
	*DKK2m1*	AGAGTTAAATCGTCGAGATTTC	146	40	60
	*DKK2m2*	CTAAAAACAATCAAATACGAAACG			
	*DKK2u1*	GGAGAGTTAAATTGTTGAGATTTT	149	40	58
	*DKK2u2*	ACTAAAAACAATCAAATACAAAACA			
	*DKK3m3*	TTTCGGGTATCGGCGTTGTC	148	40	60
	*DKK3m4*	ACTAAACCGAATTACGCTACG			
	*DKK3u3*	GTTTTTTTGGGTATTGGTGTTGTT	135	40	58
	*DKK3u4*	CAACTAAACCAAATTACACTACA			
	*DACT1m3*	CGGGATAGTAGTAGTCGGC	118	40	60
	*DACT1m4*	CGCTAAAACTACGACCGCG			
	*DACT1u3*	GTTGGGATAGTAGTAGTTGGT	123	40	58
	*DACT1u4*	AAACACTAAAACTACAACCACA			
	*DACT2m3*	CGTGTAGATTTCGTTTTTCGC	200	40	60
	*DACT2m4*	CCGAAAATCCGCCCGACG			
	*DACT2u3*	TGTGTGTAGATTTTGTTTTTTGT	203	40	58
	*DACT2u4*	CCCCAAAAATCCACCCAACA			
	*SFRP1m1*	TGTAGTTTTCGGAGTTAGTGTCGCGC	126	40	60
	*SFRP1m2*	CCTACGATCGAAAACGACGCGAACG			
	*SFRP1u1*	GTTTTGTAGTTTTTGGAGTTAGTGTTGTGT	137	40	58
	*SFRP1u2*	CTCAACCTACAATCAAAAACAACACAAACA			
	*SFRP2m1*	GGAGTTTTTCGGAGTTGCGC	128	40	60
	*SFRP2m2*	CTCTTCGCTAAATACGACTCG			
	*SFRP2u1*	GTTGGAGTTTTTTGGAGTTGTGT	133	40	58
	*SFRP2u2*	CTCTCTTCACTAAATACAACTCA			
BGS	*TET1BGS1*	TTGTTTTTTTATTGTGGATTTTTG	384	40	60
	*TET1BGS2*	AACCCACCCCTAAAACAAC			
Chip-PCR	*DACT2F*	CGTGCAGACCCCGCCCTC	113		60
	*DACT2R*	GATCCCGAGCTGTGTCGCG			
	*SFRP2F*	TGTCCCGCTTCTCCGCG	98		60
	*SFRP2R*	GAGTTCGAGCTTGTCCCG			
	*Wnt5AF*	CTCTCCGTGGAACAGTTGC	136		60
	*Wnt5AR*	GCAGAGCTGGGATGCGC			

### Bisulfite genomic sequencing and methylation-specific PCR

Bisulfite modification of DNA and methylation-specific PCR (MSP) were performed as previously described [32, 33]. Bisulfite-treated DNA was amplified with primers specific for methylated or non-methylated TET1. For bisulfite genomic sequencing (BGS), bisulfite-treated DNA was amplified using the primers shown in Table 1. The PCR products were cloned into eight to ten randomly chosen colonies using pCR4-Topo vector (Invitrogen Corporation, Carlsbad, CA) and then sequenced.

### Construction of plasmids and stable cell lines

The TET1 plasmid contains the catalytic domain (CD) including the Cys-rich and DSBH regions. Both pcDNA3.1-Flag-HA-TET1-CD-His (pcDNA3.1-TET1-CD) plasmid with the CD regions (enzymatically active) and pcDNA3.1-Flag-HA-TET1-CD-mut-His (pcDNA3.1-TET1-CD-mut) plasmid with two amino acid substitutions in CD regions (enzymatically inactive) were gifts from Prof. Qian Tao (Chinese University of Hong Kong, Hong Kong, China) [29]. pN3myc-TET1-CD (pcDNA3.1-myc-TET1) was constructed by EcoRI and XbaI enzyme digestion pcDNA3.1-Flag-HA-TET1-CD-His and subcloned into pcNDA3.1-N3myc plasmids, which was validated by RT-PCR and sequencing. And pTopflash and pFopflash were used in our previous work [34].

pcDNA3.1 vector and pcDNA3.1-TET1-CD, pcDNA3.1-TET1-CD-mut were transfected into cells using Lipofectamine2000 (Invitrogen Corporation, Carlsbad, CA, USA) according to the manufacturer’s protocol. Stable cells were confirmed by RT-PCR and Western blot.

### Dot-blot analysis

Genomic DNA was purified, sonicated, denatured in 2 × DNA denaturing buffer, and incubated at 95 °C for 10 min before spotting onto polyvinylidene difluoride (PVDF; Bio-Rad, Hercules, CA, USA) membranes. Equal amounts of DNA were allowed to spotting onto the membranes, which were then dried at room temperature. After UV cross-linking at 1200 J/m^2^ and 2 h at 60 °C in incubator, membranes were blocked with 5% low-fat milk for 1 h at room temperature and incubated with primary antibodies against 5-hmC (1:5000; #39769; active motif), 5mC (1:1000; #28692;Cell Signaling Technology) overnight at 4 °C with gentle agitation. Membranes were incubated with secondary antibodies and read to detect DNA using enhanced chemiluminescence (ECL; Amersham Pharmacia Biotech, Piscataway, NJ, USA) at last.

### Colony formation assay

Cells were stably transfected with pcDNA3.1, pcDNA3.1-TET1-CD, or pcDNA3.1-TET1-CD-mut plasmids and plated at 400 or 800 cells/well in six-well plates for HNE1 and HONE1, respectively. Following selection for 10 days with G418, colonies (≥ 50 cells/ colony) were stained with gentian violet (ICM Pharma, Singapore) and counted. All experiments were performed three times.

### Cell proliferation assay

Stably transfected HNE1 and HONE1 cells were collected, counted, and plated in 96-well plates. Proliferation was assayed after 24, 48, and 72 h by the MTS Reagent (Promega, Madison, WI, USA) (absorbance 490 nm). All experiments were performed three times.

### Wound healing and transwell assays

Stably transfected TET1-CD and vector plasmid-transfected HNE1 and HONE1 cells were cultured in six-well plates until confluent. The monolayers were scratched, washed with PBS, and cultured in 0% FBS-RPMI 1640. Cells were photographed at 0, 12, and 24 h at × 100 magnification by light microscopy (Leica DMI4000B, Milton Keynes, Bucks, UK). All experiments were performed three times.

Cell migration and invasion were evaluated in 8-μm pore size Transwell chambers (Corning Life Sciences, Corning, NY, USA). The Transwell membranes were pre-coated with Matrigel (BD Biosciences) for the invasion assay. Cells stably expressing empty-vector or TET1-CD were washed twice in PBS and serum-free medium and plated into Transwell chamber inserts in 24-well plates after counting. The lower chambers contained 700 μL culture medium with 20% FBS. After incubation in FBS-free RPMI 1640 for 24 h, the cells that had migrated into the lower chamber were fixed in 4% paraformaldehyde for 30 min and stained with 0.1% crystal violet for 30 min. The nonmigrating cells in the upper chamber were removed, and the stained cells in three randomly selected fields were photographed by × 100 magnification and counted. All experiments were performed three times.

### Flow cytometry analysis and apoptosis assay

For cell cycle analysis, HNE1 and HONE1 cells were seeded in six-well plates and transfected with 4 μg of TET1-CD or empty-vector control plasmids using lipofectamine 2000 (Invitrogen Corporation, Carlsbad, CA, USA) following the manufacturer’s protocol. After 48 h, cells were harvested, washed, fixed in ice-cold 70% ethanol overnight at 4 °C, and treated with 100 μL of 50 mg/L propidium iodide (PI; BD Pharmingen, San Jose, CA, USA) for 30 min at 4 °C in the dark. Data were analyzed with CELL Quest software (BD Biosciences, San Jose, CA, USA). Annexin V-fluorescein isothiocyanate (FITC; BD Pharmingen) and PI double staining were used for apoptosis analysis. Briefly, the transfected cells were washed with PBS, stained with AnnexinV-FITC and PI for 5 min, and visualized immediately by flow cytometry analysis. The percentage of apoptotic cells was then calculated. All experiments were performed three times.

### Western blot assay

Transfected cells were washed with ice-cold PBS and lysed using protein extraction reagent (Thermo Scientific, Rockford, IL, USA) containing phenylmethylsulfonyl fluoride and a protease inhibitor cocktail (Sigma-Aldrich, St Louis, MO). The lysate was centrifuged at 4 °C for 10 min at 10,000*g*, the liquid supernatant was collected, and 40 μg protein lysate aliquots were separated by sodium dodecyl sulfate-polyacrylamide gel electrophoresis (SDS-PAGE) and transferred to PVDF membranes (Bio-Rad, Hercules, CA, USA). The membranes were blocked with 5% low-fat milk for 1 h and subsequently incubated with primary antibodies (dilution 1:1000) overnight at 4 °C followed by incubation with an anti-mouse IgG or anti-rabbit IgG secondary antibody. The primary antibodies were Myc-tag (#2276; Cell Signaling Technology), active β-catenin (#19807s; Cell Signaling Technology), total β-catenin (#9562; Cell Signaling Technology), c-Myc (#13987s; Cell Signaling Technology), and cyclin D1 (#1677-1; Epitomics); β-actin (ARG62346; arigo) was used as a control. Proteins were visualized using an enhanced chemiluminescence (ECL) kit (Amersham Pharmacia Biotech, Piscataway, NJ, USA).

### Immunofluorescence staining

Cells were incubated on coverslips in 24-well plates and transfected with TET1-CD. After 48 h, cells were fixed in paraformaldehyde and processed for double-label immunofluorescence by incubating with HA-tag (#3724; Cell Signaling Technology), Myc-tag (#2276; Cell Signaling Technology), total β-catenin (#2677; Cell Signaling Technology), or active β-catenin (#19807s; Cell Signaling Technology) primary antibodies at 4 °C overnight and fluorochrome-labeled secondary antibodies against mouse or rabbit IgG in the dark condition. Cells were then stained with 4′, 6-diamidino-2-phenylindole (DAPI) and subsequently visualized by a confocal laser scanning microscope and photographed.

### Dual-luciferase reporter assay

Cells stably transfected with vector control and TET1-CD were plated into 24-well plates and transiently transfected with TOP/FOP-flash and Renilla luciferase reporter plasmids. The vector was the control and Renilla luciferase reporter phRL-TK was the internal control. After 48 h, cells were lysed in lysis buffer for 15 min at room temperature with shaking. Lysates were centrifuged, the supernatant was extracted, and luciferase activity was detected using a dual-luciferase reporter assay kit (Promega).

### Immunohistochemistry assay

Hematoxylin and eosin (H&E) staining is used to identify normal septum deviation and cancerous tissue morphology. Immunohistochemistry (IHC) was performed using an UltraSensitive SP Kit (Maixin-Bio, Fujian, China) following the manufacturer’s instructions. Normal septum deviation and NPC tissues were formalin-fixed and paraffin-embedded. Sections were deparaffinized, and following antigen retrieval by heating in a microwave in pH 6.0 sodium citrate solution, sections were incubated with TET1 (1:150; GTX124207; Gene Tex) and 5-hmC (1:150; #39769; active motif) primary antibodies at 4 °C overnight. Following incubation with secondary antibodies, and DAB and hematoxylin staining, cells were evaluated using Image-Pro Plus, version 6.0 (IPP6.0, Silver Spring, MD, USA).

The universal German semi-quantitative scoring system [35] was used to assess TET1 protein expression by the intensity of nuclei staining and number of stained cells. Briefly, each sample selected three to five random fields of vision at × 400 magnification, through the score of staining intensity(0 = no; 1 = weak; 2 = moderate; 3 = intense) was multiplied by the score of the range of stained cells (0 = 0%; 1 = 1~24%; 2 = 25~49%; 3 = 50~74%; 4 = 75~100%) to give the score of one field of vision, and then taking the average (range from 0 to 12). The scores for immunostaining were scored by two individuals who did not know the sample information. Student’s *t* test was used for statistical analysis.

### Methylated DNA immunoprecipitation (MeDIP) and Hydroxymethylated DNA immunoprecipitation (hMeDIP)

Two micrograms of sonicated DNA was denatured at 95 °C for 10 min, immediately cooled on ice for 10 min, and diluted in 500 μl of IP buffer (10 mM Na-Phosphate pH 7.0 140 mM NaCl 0.05% Triton X-100), 50 μl as input. Then, add 10 μl of anti-5mC (#28692; Cell Signaling Technology) or anti-5hmC (#39769; active motif; Cell Signaling Technology), IgG (#2729, Cell Signaling Technology) as control. After 2 h incubation at 4 °C with overhead shaking, 30 μl of 50% protein A/G-Sepharose magnetic beads were added and incubated for another 2 h. Put the tube on the magnetic beam for 5 min and discard supernatant. After five washes with IP buffer, DNA was eluted with Proteinase K for 2 h, then purified using the QIAQuick PCR Purification Kit (Qiagen) according to the manufacturer’s instructions. DNA was analyzed by quantitative real-time PCR by using a SYBR GreenER kit (Invitrogen). Primers sequences are provided in Table 1. Fold enrichment was calculated as follows: %Input = 10% × 2^(CT^input^−CT^sample^).

### Statistical analysis

Continuous variables were reported as the mean value ± standard deviation (SD) compared by Student’s *t* test. Categorical values were compared by the chi-square test or Fisher’s exact test. Differences accepted for significance was *p* < 0.05. All data analyses were carried out with the statistical analysis software package SPSS22.0 (SPSS Inc., Chicago, IL, USA). All biostatistics calculations were performed using Prism (Graphpad Software, Inc., La Jolla, California).

## Results

### TET1 expression is downregulated in NPC cell lines

**Fig. 1:**
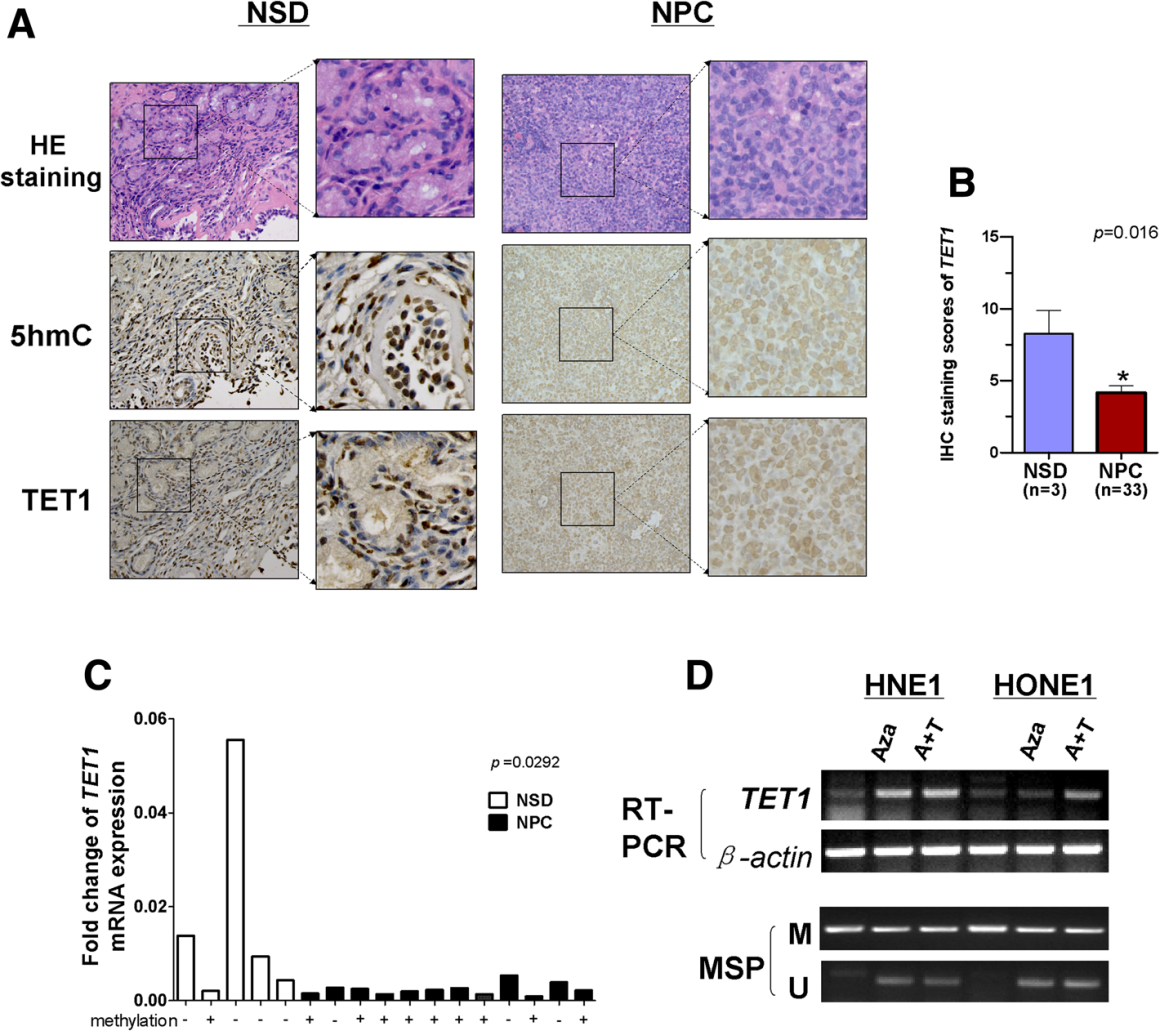
Expression of TET1 protein and mRNA in normal septum deviation tissues (NSD) and NPC. **a** Representative IHC images of TET1 and 5-hmC expression in normal septum deviation tissue and NPC tissue. The staining scores of TET1 expression was shown in (**b**). **c** TET1 mRNA expression in normal septum deviation tissue and NPC assayed with qRT-PCR, β-actin was used as a control. Results are means ± SD, *p* = 0.0292 (NPC), *p* = 0.0088(HNSC). **d** Pharmacological demethylation of TET1 CGI by AZA (A) with or without TSA (T) induced its expression. *TET1* expression before and after drug treatment was determined with RT-PCR, and demethylation was confirmed with methylation-specific PCR

Our previous studies found that TET1, 2, and 3 are expressed in human adult and fetal tissues and that only TET1 was downregulated in NPC cell lines [29]. H&E staining showed normal epithelial and cancer cell morphology and IHC staining showed that both TET1 and 5hmC expression were higher in 3 normal septum deviation than in 33 NPC tissues (Fig. 1a, b). Unfortunately, there was no correlation between TET1 expression and clinical and pathological features (Table 2). qRT-PCR was used to assay *TET1* mRNA expression and revealed that it was higher in normal nasal tissues than NPC (*p* = 0.0292), and most samples with low TET1 expression show considerably high methylation in CpG island of TET1 promoter (Fig. 1c). When methylated or silenced HNE1 and HONE1 nasopharyngeal cells were treated with the demethylation reagent 5-aza-2′-deoxycytidine (Aza) with or without trichostatin A (TSA) histone deacetylase, PCR confirmed that *TET1* expression was restored with a significant decrease of *TET1* promoter methylation (Fig. 1d). The results indicate that CpG methylation of *TET1* promoter mediated its silencing in NPC cells.

**Table 2:** Association between TET1 expression and clinical characteristics in NPC tissues

Characteristics	Number (*n* = 33)	TET1 expression				*p* value
		None	Low	Moderate	High	
Age (years)						
≧ 48	16	2	9	4	1	0.778
< 48	17	1	8	6	2	
Gender						
Female	9	0	4	4	1	0.548
Male	24	3	13	6	2	
WHO histological type						
I (keratinizing)	5	0	2	3	0	0.401
II/III (non-keratinizing)	28	3	15	7	3	
Clinical stages						
I	6	1	4	1	0	0.255
II	13	1	8	4	0	
III	12	1	5	3	3	
IV	2	0	0	2	0	
Lymph node metastasis						
positive	21	1	9	8	3	0.179
negative	12	2	8	2	0	

### TET1 promoter is frequently methylated in NPC tissues

**Fig. 2:**
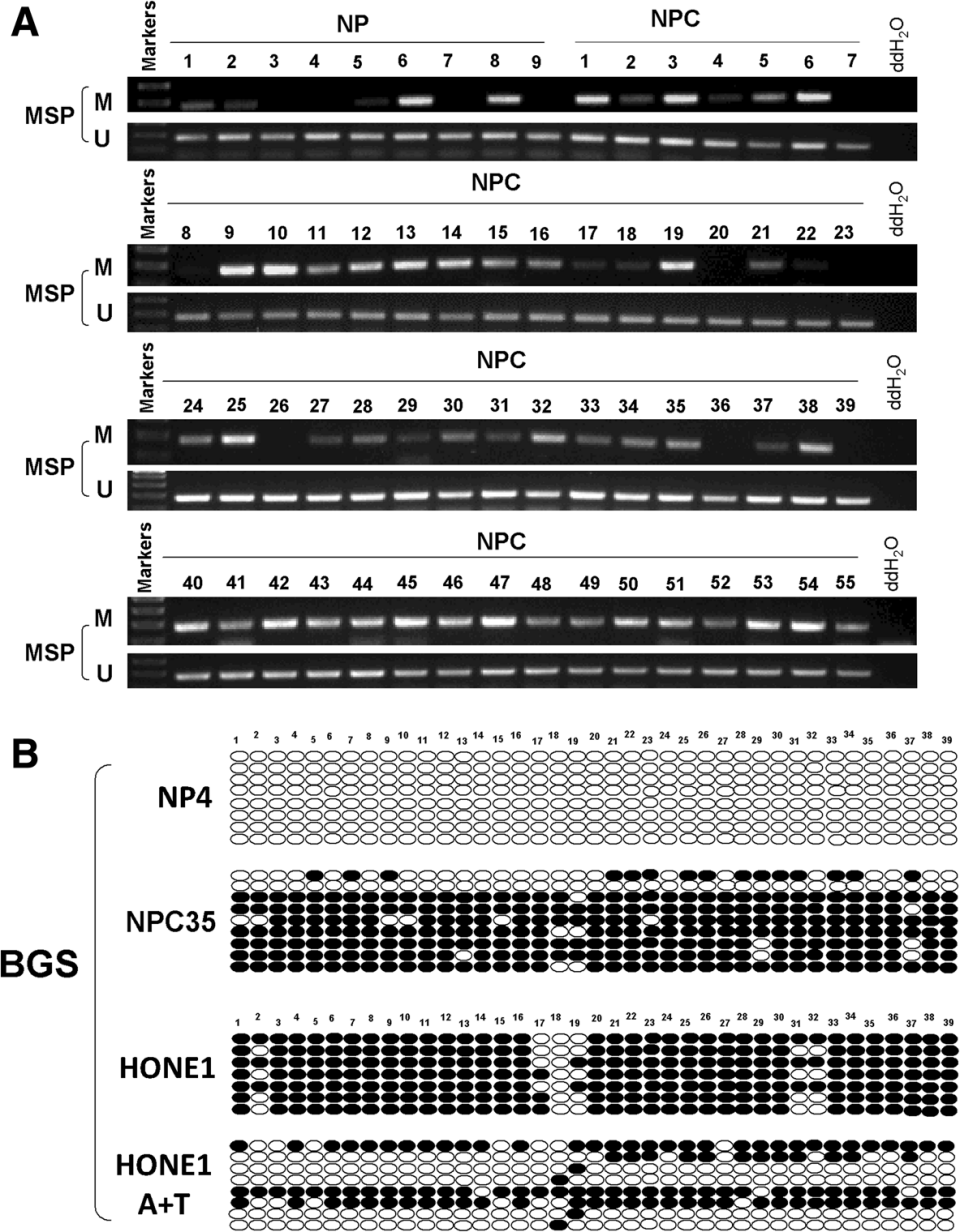
The methylation status of *TET1* in NSD, surgical margin tissues, nasopharyngeal carcinoma (NPC) tissues, and nasopharyngeal carcinoma cells lines. **a** Promoter methylation of *TET1* in surgical margin tissues and nasopharyngeal carcinoma tissues as measured by MSP. M: methylated; U: unmethylated. **b** Bisulfite genomic sequencing confirmed the methylation status of *TET1* CpG sites in NSD, NPC tissues, and HONE1 cells with or without A+T treatment

The CpG islands of TET1 and primer design for MSP have been shown in previous study [29]. Methylation-specific PCR was used to assay 55 NPCs and 9 surgical margin tissue samples. Hyper-methylation of the *TET1* promoter was observed in 45/55 (81.8%) NPCs and 4/9 (44.4%) in surgical margin tissues, indicating that methylation of the TET1 promoter was a common event in NPC (Fig. 2a). BGS analysis further confirmed the MSP data (Fig. 2b).

### Ectopic expression of TET1 suppresses NPC cell growth mediated by its catalytic domain

**Fig. 3:**
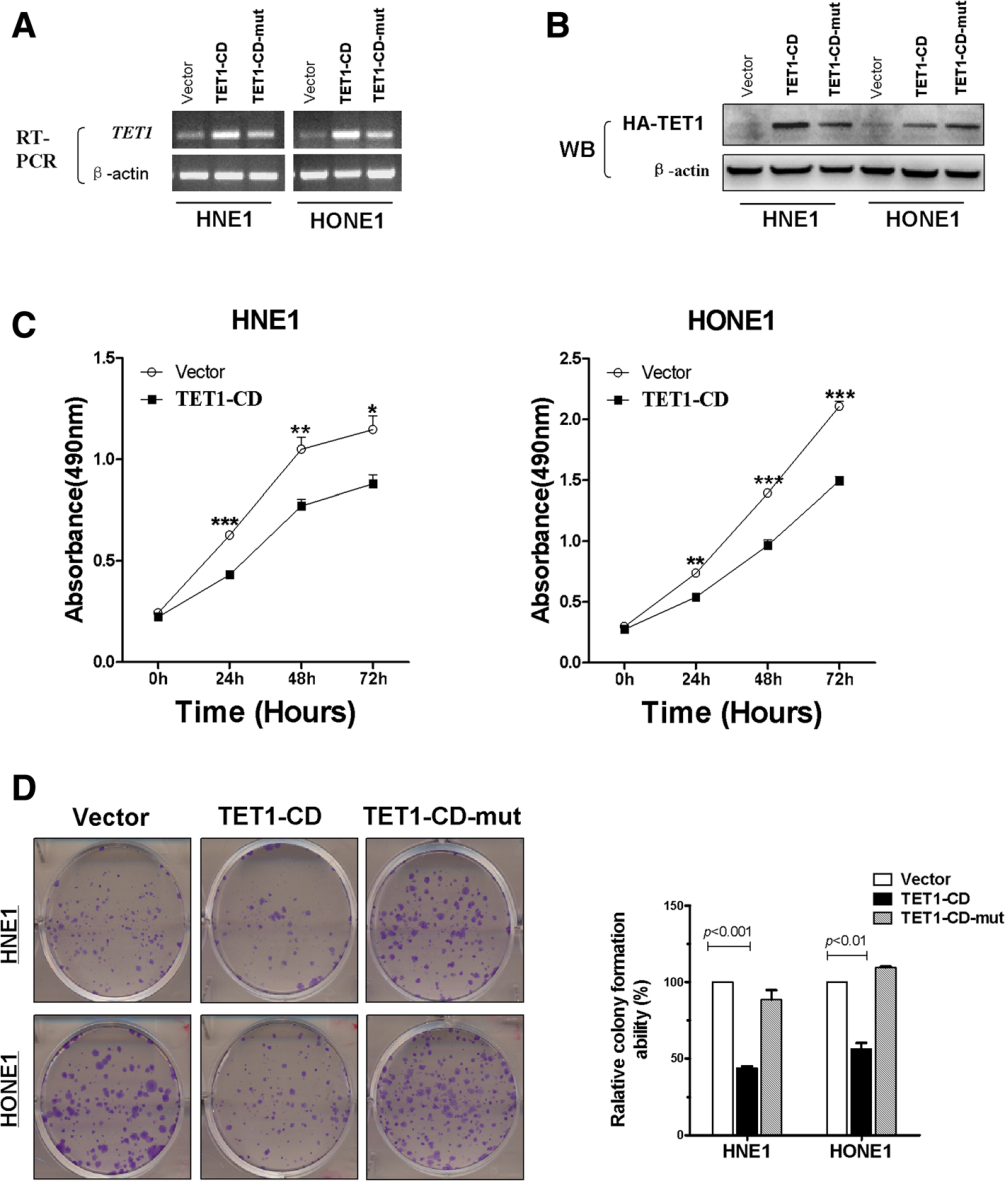
TET1 suppresses NPC cells proliferation via its catalytic domain. **a**, **b** RT-PCR and Western blots show TET1 expression in vector controls, TET1-CD- and TET1-CD-mut-transfected HNE1 and HONE1 cells. **c** The proliferation assay of vector controls and TET1-expressing HNE1 and HONE1 cells. *p < 0.05, **p < 0.01, ***p < 0.001 (**d**). Colony formation assay of HNE1 and HONE1 cell transfected with Vector, TET1-CD and TET1-CD-mut

As TET1 was downregulated in NPC and expressed in normal septum deviation, the tumor repressive of TET1 overexpression on the growth of NPC cells was evaluated. RT-PCR and Western blotting confirmed the expression of TET1 mRNA and protein in stably transfected HNE1 and HONE1 cells, which had few intrinsic TET1 expressions (Fig. 3a, b). MTS and colony formation assays revealed that TET1 significantly suppressed cell viability at 24, 48, and 72 h (*p < 0.05, **p < 0.01, and ***p < 0.001, respectively) and decreased colony formation by 45–55% compared with cells transfected with empty vectors, but no difference between empty vector group and TET1-CD-mut group, i.e., with inactive catalytic domains (Fig. 3c, d). The results indicated that re-expression of TET1 with an active catalytic domain suppressed the growth of NPC cell lines.

### TET1 induces NPC cell cycle arrest in G0-G1 phase and apoptosis

**Fig. 4:**
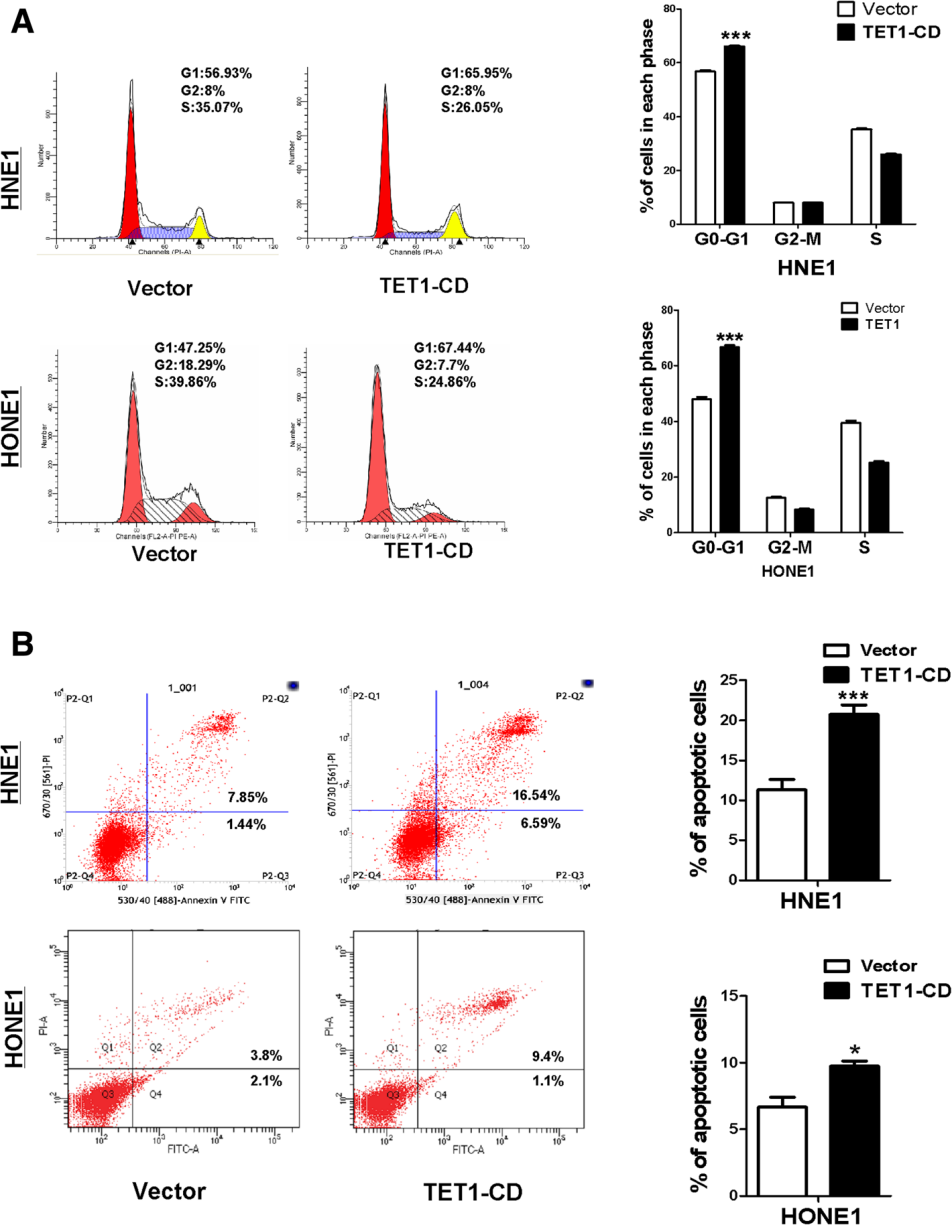
TET1 induces cell cycle arrest at G0/G1 phase and induces cell apoptosis. **a** Left: Representative cell cycle distribution vector- and TET1- tranfected HNE1 and HONE1 cells by flow cytometry analysis. Right: Data summary (***p < 0.001). **b** Apoptotic cells are double-stained with Annexin V-FITC and PI. Cells were indicated as in **a**

Flow cytometry was used to determine whether TET1 affected the cell cycle and apoptosis of NPC cells. TET1-transfection resulted in a 20 and 9% increase of the numbers of HNE1 and HONE1 cells in the G0/G1 phase (*p* < 0.001) compared with controls, respectively (Fig. 4a). Transfection also increased the number of apoptotic cells (Fig. 4b). These results demonstrated that TET1 inhibited the proliferation of NPC cells by inducing cell cycle arrest and promoted apoptosis.

### TET1 suppresses NPC cells migration and invasion via regulating epithelial-mesenchymal transition

**Fig. 5:**
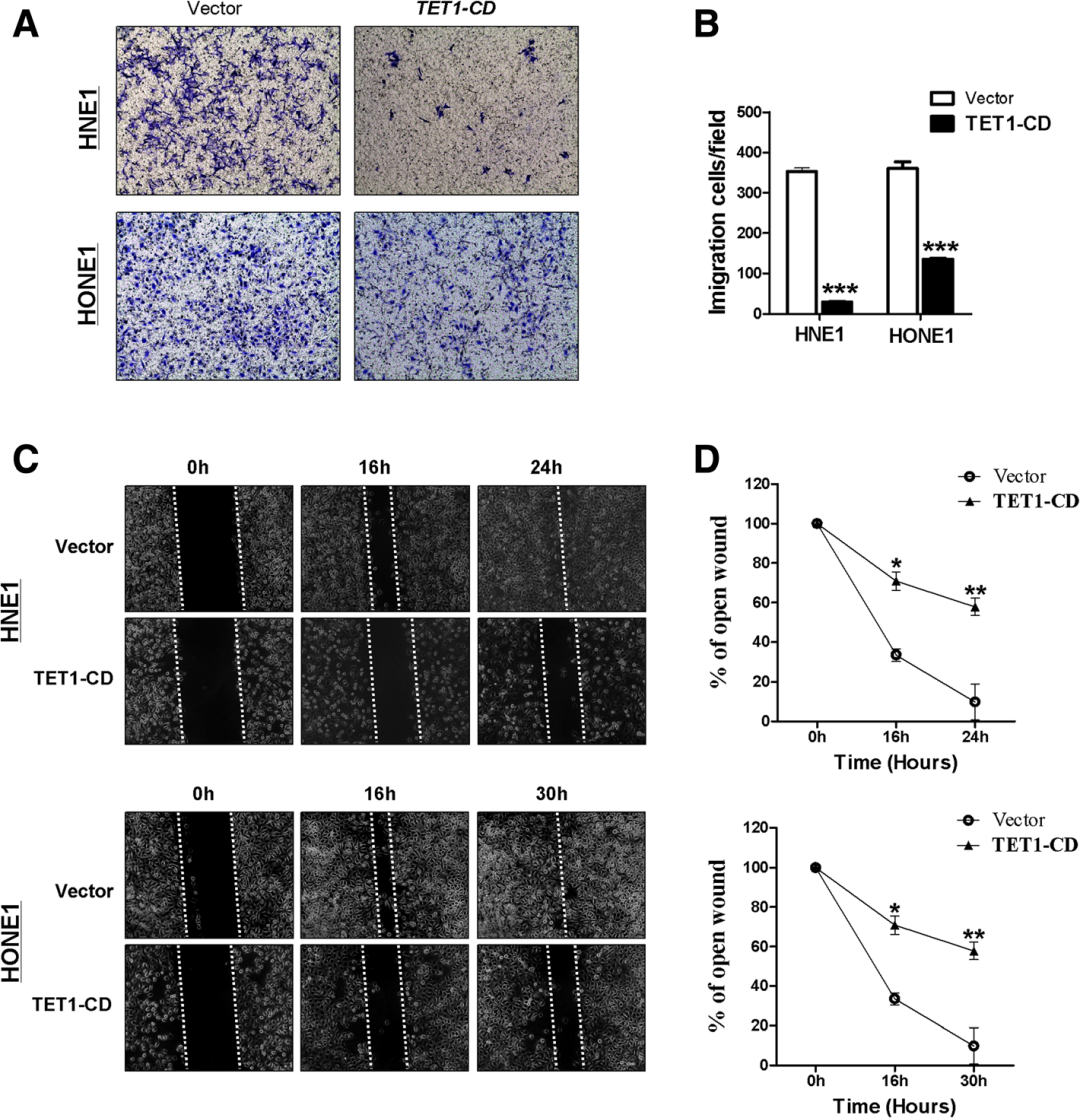
TET1 suppresses HNE1 and HONE1 cells migration. **a** Transwell assay shows the migration of cells transfected with vector or TET1. Photographs show cells that crossed the membrane; the numbers of cells are shown in **b** (***p < 0.001). **c** Wound healing assay evaluated cell migration at 0, 16, 24, and 30 h. The wound healing rate was calculated in **d**

**Fig. 6:**
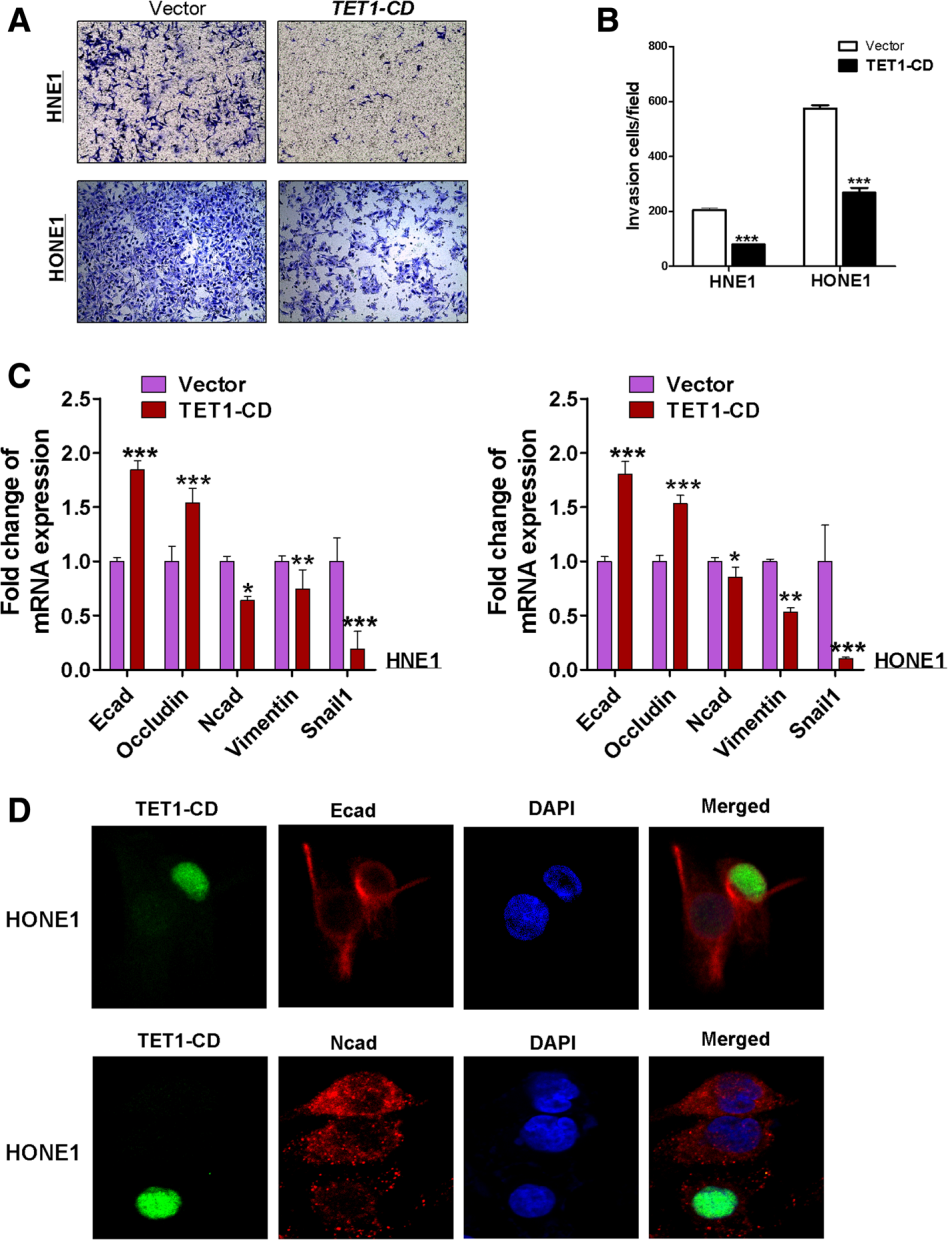
TET1 suppressed NPC cells invasion by inhibiting EMT process. **a** Transwell assay of cell invasiveness showing cells that traversed the Matrigel-coated membrane. The analysis is shown in (**b**). **c** The expression of E-cadherin, N-cadherin, Occuldin, Vimentin, and snail1 in TET1-CD- expressing HNE1 and HONE1cells was determined by qRT-PCR. **d** Subcellular location and expression of E-cadherin and N-cadherin in HONE1 cells by immunofluorescence staining

Transwell chamber motility showed that motility was significantly suppressed in HNE1 and HONE1 cells expressing TET1 (Fig. 5a, b). Similar results were observed in wound healing assays (all *p* < 0.001) (Fig. 5c, d). Matrigel invasiveness assays demonstrated that ectopic TET1 expression significantly inhibited HNE1 and HONE1 cell invasion in culture medium containing 20% FBS (Fig. 6a, b). These results indicated that TET1-expression inhibited migration and invasion of NPC cells. The epithelial to mesenchymal transition (EMT) plays an important role in cancer progression and metastasis. To verify whether TET1 suppresses cell migration caused by inhibiting EMT, the expression of epithelial and mesenchymal markers was investigated. qRT-PCR showed the increase of E-cadherin and occludin and the decrease of N-cadherin, vimentin, and snail1 in TET1-expressed HNE1 and HONE1 cells (Fig. 6c). And immunofluorescence staining proved the same results in HONE1 cells (Fig. 6d). These results suggested that TET1 inhibits EMT in NPC cells.

### TET1 inhibits the Wnt/β-catenin signaling pathway

**Fig. 7:**
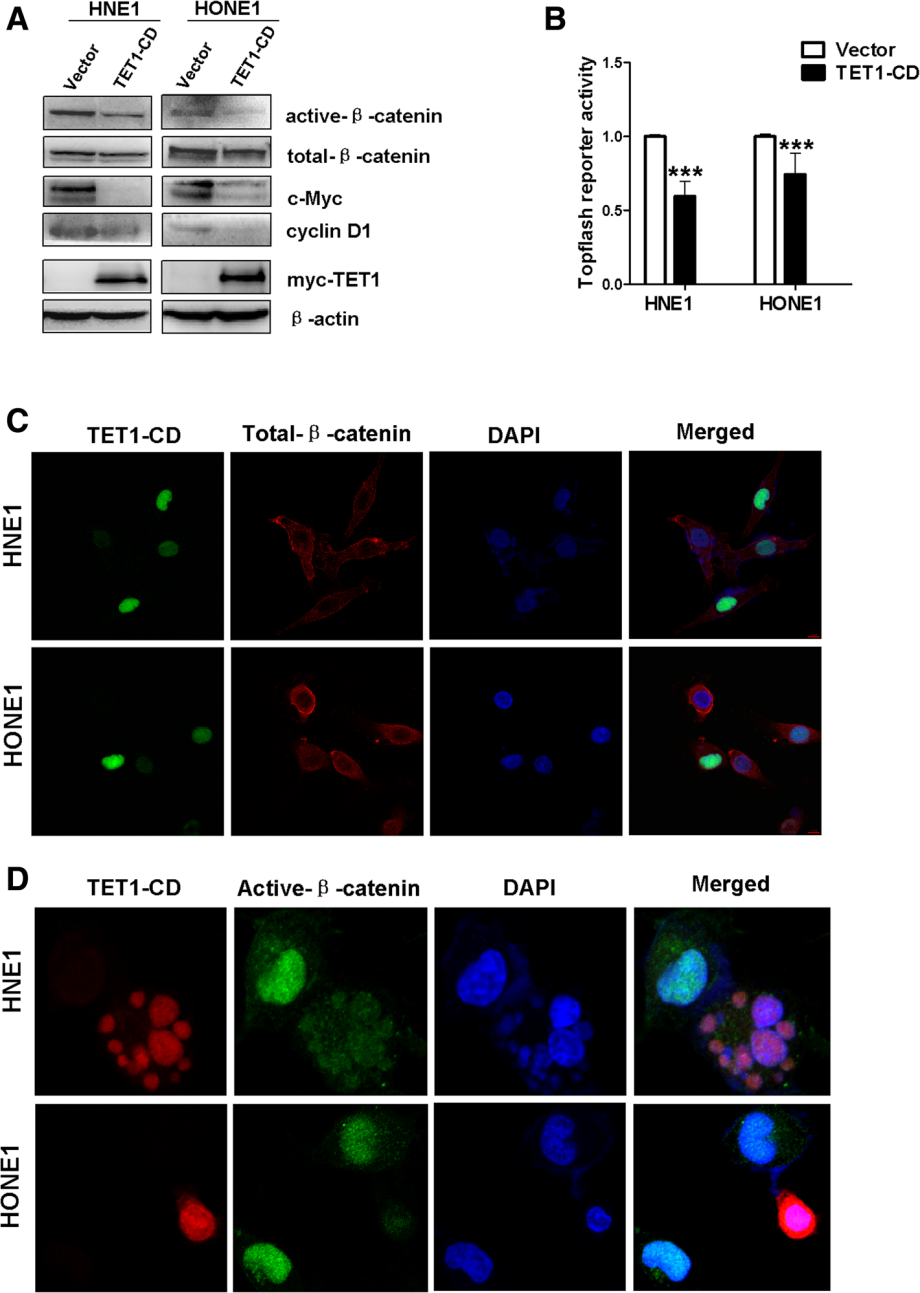
TET1 inhibits Wnt/β-catenin signaling pathway in NPC. **a** Western blots of β-catenin and the downstream Wnt/β-catenin gene. **b** Top/Fop dual-luciferase reporter assay in HNE1 and HONE1 cells. Fop-flash was the control. **c**, **d** Subcellular location and expression of total β-catenin and active β-catenin by immunofluorescence staining

Aberrant activation of the Wnt/β-catenin signaling pathway was involved in NPC carcinogenesis. The evidence indicated that methylation of genes promoting negative Wnt regulators contributes to aberrant silencing and activation of Wnt/β-catenin signaling in human cancers. Western blotting, TOP/FOP-Flash, and immunofluorescence were used to investigate the effect of TET1 on Wnt/β-catenin signaling in NPC cells. The expression of β-catenin and some downstream target genes of Wnt/β-catenin were confirmed in NPC cells. Active β-catenin, c-Myc, and cyclinD1 were downregulated, with no significant change in total β-catenin in NPC cells overexpressed TET1 (Fig. 7a). TOP-flash assays found that TET1 overexpression had a significant inhibitory effect on β-catenin/TCF activity compared with FOP-flash controls (Fig. 7b). Immunofluorescence confirmed that nuclear expression of active β-catenin was decreased by TET1 overexpression, and total β-catenin has no significant change (Fig. 7c, d). Collectively, the data demonstrated that TET1 inhibited the Wnt/β-catenin signaling pathway in NPC cells.

### TET1 renews expression of Wnt antagonists by demethylation of their promoter

To determine how TET1 influenced Wnt/β-catenin signaling, the expression of Wnt pathway antagonists, including DACT (DACT1,2,3), SFRP (SFRP1,2,3), DKK (DKK1-3) family genes, and WNT proteins (include WNT1,3,3A,4, WAN5A, WNT5B, WNT7A, WNT7B) in HNSN (head and neck squamous cell normal tissues) and HNSC (head and neck squamous cell carcinoma) was analyzed. DACT (1,2,3), WNT5A, and WNT7B were significantly positive correlation with TET1(*p* < 0.05), and WNT4 and WNT7A were negative correlation with TET1 in the TCGA Head and Neck Squamous cell Carcinoma database (*p* < 0.05, Additional file 1: Figure S1). We further found that *DACT2* and *WNT5B* had the low-expression and hypermethylation in HNSC compared with HNSN (Additional file 2: Figure S2), data from TCGA (http://methhc.mbc.nctu.edu.tw/php/index.php). Based on above information, DACT (1,2,3), WNT5A, WNT5B, and WNT7B were most likely to be affected by TET1 in NPC.

**Fig. 8:**
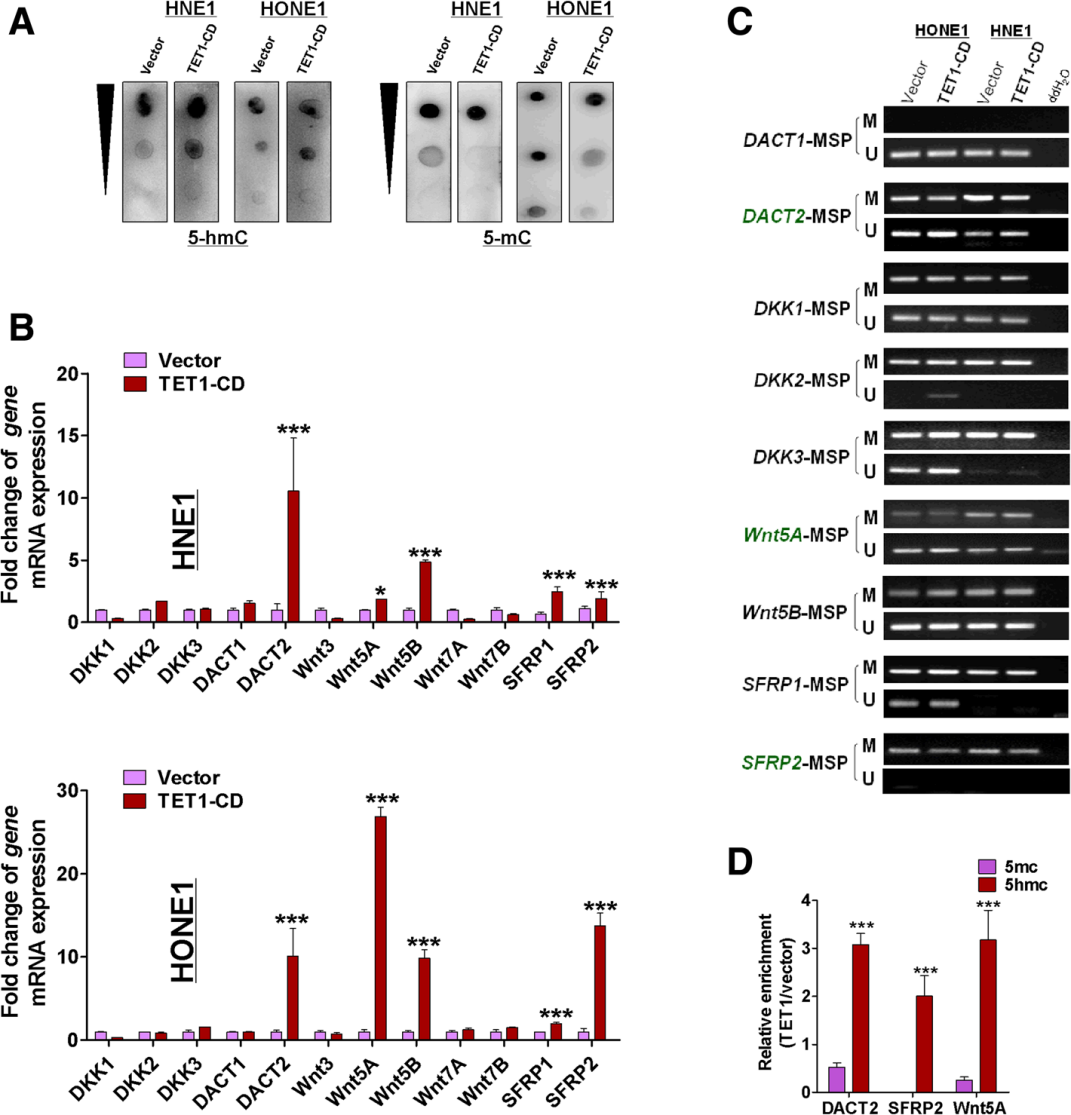
TET1 restored the expression of Wnt antagonists by demethylation of their promoter. **a** Dot-blot assay of 5-hmC and 5-mC in HNE1 and HONE1 cells with DNA concentration gradients. **b** The expression of target genes (DKKs, DACTs, SFRPs, and WNTs) in vector- and TET1-CD-transfected NPC cells was detected by qRT-PCR. **c** Methylation of *DACTs*, *DKKs*, *SFRPs*, and *WNTs* by MSP in vector- and TET1-transfected HNE1 and HONE1 cells. **d** The enrichment of 5-hmC and 5-mC in the promoter of *Wnt5A*, *DACT2*, and *SFRP2* was detected by MeDIP-qPCR and hMeDIP-qPCR in HNE1 cells

Dot-blot assays were used to characterize 5hmC and 5mC expression in NPC cell lines that over-expressed TET1. TET1 re-expression in transfected TET1-silenced cells resulted in increased levels of 5hmC and decreased levels of 5mC compared with empty-vector transfected cells (Fig. 8a). To determine how TET1 influenced Wnt/β-catenin signaling, the expression of Wnt pathway antagonists, including DACT family genes (*DACT1*, *2*, *3*), DKK (*DKK1*, *2*, *3*) family genes, and SFRP family genes (*SFRP1* and *SFRP2*), in TET1-transfected and vector-transfected cells was assayed by qRT-PCR and revealed an increase of *DACT2*, *SFRP1*, and *SFRP2* mRNA expression in TET1-transfected cells compared with vector controls (Fig. 8b). In addition to Wnt pathway antagonists, we also found that the *Wnt5A* and *Wnt5B* were upregulated in TET1-transfected NPC cells (Fig. 8b). Furthermore, we found that Wnt5B, SFRP1, and SFRP2 were hypermethylated in nasopharyngeal carcinoma tissues (Additional file 3: Figure S3), and the hypermethylation of DKKs and DACTs in nasopharyngeal carcinoma tissues was also confirmed [15, 36]. In the meantime, the reduced methylation status of DACT2, Wnt5A, and SFRP2 promoter was detected in TET1-CD-expressing tumor cells, with increased unmethylated sites at the promoter CpG regions (Fig. 8c), suggesting that TET1 really acts as a demethylase to renew expression of multiple TSGs in tumor cells. MeDIP and hMeDIP experiments were used to further confirm the demethylation of TET1; the results showed that TET1 can cause an increase of 5-hmC and reduction of 5mC in promoters of Wnt5A, SFRP2, and DACT2 in HNE1 cells (Fig. 8d). These studies suggested that TET1 can cause demethylation of Wnt5A, SFRP2, and DACT2 in nasopharyngeal carcinoma cells and restore their expression.

## Discussion and conclusion

TET1 has been described as a TSG, inhibiting proliferation in renal, colorectal, and gastric cancer [24, 25, 28], and migration and invasion in the lung, breast, and prostate cancer [26, 37, 38]. This study found that TET1 was expressed in normal septum deviation and downregulated in NPC tissues as well as NPC cells, and it was methylated in most NPC tissues and cells. This implies that *TET1* was silenced by hyper-methylation in NPC. Demethylation by Aza with TSA can restore TET1 expression, but HONE1 treated only by Aza did not restore TET1 expression, indicating other mechanism involving TET1 expression. In present study, TET1 suppressed NPC cell proliferation, motility, and invasiveness. It also induced G0/G1 phase arrest and apoptosis. The results were consisted with other cancers. So, our data indicate that TET1 acts as a TSG activity in NPC.

Many TSGs act by inhibiting Wnt/β-catenin signaling, a pathway known to be sensitive to TSG methylation in NPC [15, 16, 39–41], and TET1 was previously shown regulating the Wnt/β-catenin pathway in colorectal cancer [24]. We found that TET1 inhibits the expression of β-catenin and its target genes, cyclinD1 and c-Myc. TET1 also prevented β-catenin interaction with T-cell factor/lymphoid enhancer factor (TCF/LEF) transcription factors, which are end point mediators of Wnt signaling. These data demonstrated that TET1 suppressed NPC cell growth by regulating the Wnt/β-catenin signaling pathway.

To determine how TET1 regulated the Wnt/β-catenin signaling pathway, the expression of downstream target genes was assayed. It was previously demonstrated that TET1 could demethylate the PTEN gene, which codes for phosphatase and tensin homolog protein, to inhibit tumor growth in gastric cancer [42]. TET1 suppression of matrix metalloproteins in breast cancer inhibits tumor cell invasion through maintaining expression of *TIMP*2 and *TIMP3* [37]. Epigenetic disruption of Wnt/β-catenin signaling is crucial to several tumorigeneses, especially by promoter methylation of WNT antagonists [43–45]. Methylation of *SFRPs*, *DACT2*, *DKK2*, and *DKK3* was frequently detected in NPC [15]. Recent works suggest that *TET1* upregulates DKKs, which are Wnt pathway inhibitors that suppress CRC and ovarian cancer cell proliferation [24, 46]. Given the crucial role of TET1 in epigenetic modification of WNT antagonists, we thus aimed to thoroughly analyze the expression of Wnt pathway negative regulators in TET1-transfected cells. Our data clearly demonstrated that enforced expression of TET1 indeed significantly restore the expression of *DACT2*, *SFRP1*, and *SFRP2*, but no obvious influence to *DKKs* family, *DACT1* and *DACT3*. In addition, TET1 also increased the expression of *Wnt5A* and *Wnt5B*. And we have shown that *Wnt5B*, *SFRP1*, *SFRP2*, and *DACT2* are hypermethylated in nasopharyngeal carcinoma tissues [36]. But further analysis showed that the methylation status and 5-mC levels of *DACT2*, *SFRP2*, and *Wnt5A* promoter were decreased, accompanying with 5-hmC increased after TET1 upregulation, whereas there were no obvious changes in the promoters of *Wnt5B* and *SFRP1*, suggesting that other mechanisms are involved in *Wnt5B* and *SFRP1* upregulation in TET1 transfected cell line. Li et al. have confirmed that SFRP2 function as TSG in NPC [15]. We also demonstrated that DACT2 act as a TSG in NPC [36].

The Wnt pathway includes canonical Wnt/β-catenin and non-canonical Wnt signaling, which have different roles in cancer progression [15]. The Wnts family includes 19 members. Wnt1, Wnt3, Wnt3A, Wnt7A, Wnt7B, and Wnt8 participate in β-catenin-dependent signaling; Wnt4, Wnt5A, and Wnt11 participate in β-catenin-independent signaling. The canonical Wnt protein Wnt3A promotes the secretion of β-catenin and cancer development. The noncanonical Wnt protein, Wnt5A, inhibits Wnt3A promotion of β-catenin secretion. So far, there are conflicting reports as to whether WNT5A acts as a tumor promotion genes or a tumor suppressor in cancers. Most of the evidence shows that WNT5A overexpression promotes the proliferation, invasion, and metastasis of cells in different types of cancers by promoting the epithelial-mesenchymal transition (EMT) and stem cell-like phenotypes [47–50]. However, contradictory reports showed that increased Wnt5A expression inhibited cell proliferation and motility, and negative Wnt5A expression contributes to the tumor lymph node metastasis and poor prognosis [51–54]. Recently, it was shown that the contradictory roles of Wnt5A are due to existence of various Wnt5A isoforms [55]. Further studies will be needed to identify functions of Wnt5A in NPC.

Added up, we provided the first evidence indicating that TET1 was a TSG in NPC cells and suppressed NPC proliferation, migration, and invasiveness progression via restoring Wnt pathway antagonist expression to antagonize activity of Wnt pathway. Furthermore, this study provides important insights into the regulation of TET1 for Wnt pathway.

## Additional files

Additional file 1: **Figure S1.** The correlation between TET1 and DKKs, DACTs, SFRPs, and WNTs expression respectively in HNSC. Data from TCGA (http://methhc.mbc.nctu.edu.tw/php/index.php). (TIF 74 kb)

Additional file 2: **Figure S2.** The methylation and expression of TET1, DACTs, WNTs in HNSC. Data from TCGA (http://methhc.mbc.nctu.edu.tw/php/index.php). (TIF 82 kb)

Additional file 3: **Figure S3.** Detection of *Wnt5B*, *SFRP1*, and *SFRP2* methylation in nasopharyngeal carcinoma tissues by MSP. HNE1 cells and ddH_2_O were used as positive and negative control, respectively. M: methylated; U: unmethylated. (TIF 201 kb)

## Abbreviations

5-hmC: 5-Hydroxymethyl cytosine
5-mC: 5-Methyl cytosine
AnnexinV-FITC: Annexin V-fluorescein isothiocyanate
Aza: 5-Aza-2-deoxycytidine
BGS: Bisulfite genomic sequencing
DAPI: 4, 6-Diamidino- 2- phenylindole
EMT: Epithelial-to-mesenchymal transition
MSP: Methylation-specific PCR
NPC: Nasopharyngeal carcinoma
NSD: Normal nasal tissues
PVDF: Polyvinylidene difluoride
qRT-PCR: Quantitative real-time PCR
RT-PCR: Semi-quantitative RT-PCR
SDS-PAGE: Sodium dodecyl sulfate polyacrylamide gel electrophoresis
TCF/LEF: T Cell factor/lymphoid enhancer factor
TET: Ten-eleven translocation
TET1: Ten-eleven translocation methyl cytosine dioxygenase1
TSA: Trichostatin A
